# Deaths of U.S. Citizens Undergoing Cosmetic Surgery — Dominican Republic, 2009–2022

**DOI:** 10.15585/mmwr.mm7303a3

**Published:** 2024-01-25

**Authors:** Matthew Hudson, Jose A. Matos, Bianca Alvarez, Jacqueline Safstrom, Francisco Torres, Sharmeen Premjee, Luis Bonilla, Benjamin Park, Elizabeth Bancroft, Macarena Garcia

**Affiliations:** ^1^Division of Healthcare Quality Promotion, National Center for Emerging Zoonotic and Infectious Diseases, CDC; ^2^Epidemic Intelligence Service, CDC; ^3^Dominican Republic Ministry of Health, Santo Domingo, Dominican Republic; ^4^CDC Dominican Republic Country Office, Global Health Center, CDC.

**Summary**

**What is already known about this topic?**

Infections regularly occur after cosmetic surgery performed outside the United States, but deaths are rarely reported.

**What is added by this report?**

The number of deaths after cosmetic surgery among U.S. citizens in the Dominican Republic increased from a mean of 4.1 per year during 2009–2018 to a mean of 13.0 during 2019–2022 with a peak in of 17 in 2020. A review of the 29 deaths during 2019–2020 revealed that the deaths were associated with fat or venous thromboembolism. A high proportion of patients who died had risk factors for embolism, including obesity and having multiple procedures performed during the same operation.

**What are the implications for public health practice?**

Persons interested in cosmetic surgery should discuss the risks with their regular medical professional. Public health authorities can support provider education on the importance of preoperative patient evaluation and the potential danger of performing multiple cosmetic procedures in one operation.

## Abstract

Although infections resulting from cosmetic surgery performed outside the United States have been regularly reported, deaths have rarely been identified. During 2009–2022, 93 U.S. citizens died after receiving cosmetic surgery in the Dominican Republic. The number of deaths increased from a mean of 4.1 per year during 2009–2018 to a mean of 13.0 during 2019–2022 with a peak in of 17 in 2020. A subset of post-cosmetic surgery deaths occurring during peak years was investigated, and most deaths were found to be the result of embolic events (fat emboli or venous thromboembolism) for which a high proportion of the patients who died had risk factors, including obesity and having multiple procedures performed during the same operation. These risk factors might have been mitigated or prevented with improved surgical protocols and postoperative medical care, including prophylactic measures against venous thromboembolism. U.S. citizens interested in receiving elective cosmetic surgery outside the United States should consult with their health care professionals regarding their risk for adverse outcomes. Public health authorities can support provider education on the importance of preoperative patient evaluation and the potential danger of performing multiple cosmetic procedures in one operation.

## Introduction

Traveling to another country to receive medical care (medical tourism), including travel related to cosmetic surgery, is increasingly common among U.S. residents because the cost is lower and wait times for procedures are shorter than in the United States (*1*). The Dominican Republic is popular for medical tourism because it is close to the United States, has an existing tourism infrastructure, and some doctors from the Dominican Republic advertise in the United States. Since 2003, CDC has documented adverse events occurring in U.S. citizens after cosmetic surgery in the Dominican Republic (*2*). Most reports of adverse events after medical tourism for cosmetic surgery have cited infections; deaths have rarely been reported (*1*).

Since 2009, the Consular Section of the U.S. Embassy in the Dominican Republic has recorded cosmetic surgery–associated deaths among U.S. citizens. Notices of deaths are obtained from a variety of sources including families, funeral homes, and the Instituto Nacional de Ciencias Forenses (National Institute of Forensic Sciences), which conducts autopsies on all foreign citizens who die within the Dominican Republic. During 2009–2018, a mean of 4.1 deaths in U.S. citizens who had received cosmetic surgery in the Dominican Republic occurred each year (range = 1–8 deaths); however, the number of cosmetic surgery–associated deaths increased to 12 in 2019 and 17 in 2020. Because of this increase, the U.S. Embassy contacted CDC. In collaboration with the Dominican Republic Ministry of Health (MOH), CDC launched an investigation to identify the etiology and epidemiology of and preventable risk factors for death among U.S. citizens who underwent cosmetic surgery in the Dominican Republic.

## Methods

A case was defined as a death occurring in a U.S. citizen who had received cosmetic surgery in the Dominican Republic within the preceding 3 weeks and who died in the Dominican Republic during 2009–2022.^†^ Cosmetic surgical procedures were defined as all procedures falling under the American Society of Plastic Surgeons’ designations as cosmetic surgery, such as breast procedures, abdominoplasty, liposuction, and gluteal augmentation (*3*). CDC analyzed the number of deaths collected by the Consular Section at the U.S. Embassy, conducted a review of available medical and autopsy reports provided by the MOH during 2019–2020, and extracted information on known risk factors for perioperative death. This activity was reviewed by CDC, deemed not research, and was conducted consistent with applicable federal law and CDC policy.^§^

## Results

A total of 93 cosmetic surgery–related deaths of U.S. citizens in the Dominican Republic occurred during 2009–2022; all but one occurred in women. The mean decedent age was 40 years (range = 19–69 years). The number of deaths varied from one to 17 per year, with the peak in 2020 (Figure). 

**FIGURE:**
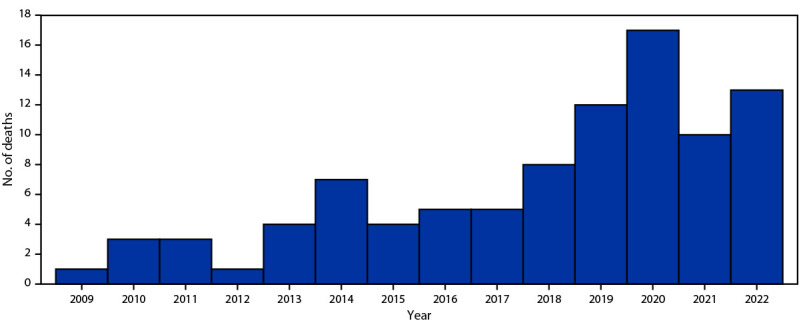
Perioperative cosmetic surgery–related deaths*^,^^†^ among U.S. citizens in the Dominican Republic, by year (N = 93) — Dominican Republic, 2009–2022 * Data provided by the U.S. Embassy Consular Section, Santo Domingo, Dominican Republic. ^†^ Deaths occurring among U.S. citizens, in the Dominican Republic, within 3 weeks of the cosmetic surgical procedure.

### Patient Characteristics

Medical records were available for 24 (83%) of the 29 deaths that occurred during 2019–2020, including 10 in 2019 and 14 in 2020 (Table). All 24 deaths occurred in women; the mean age was 41 years (range = 26–61 years), and the mean body mass index (BMI) was 32 kg/m^2^ (range = 24–44 kg/m^2^); in 22 (92%) cases, comorbid conditions associated with increased risk for venous thromboembolism were reported. Among 23 decedents with information on BMI available, 22 (96%) had overweight or obesity,^¶^ two of 24 (8%) reported diabetes mellitus, three (13%) reported current tobacco use, and two (8%) reported current use of oral contraceptives. No other patient-specific risk factors for venous thromboembolism were identified. A preoperative evaluation by a cardiologist was documented for 18 (75%) of the 24 decedents and by a pulmonologist for 11 (46%).

### Procedures

Liposuction was performed in all 24 fatal cases (100%), gluteal fat transfer in 22 (92%), abdominoplasty in 14 (58%), and breast augmentation in 11 (46%). A mean of three procedures (range = two to four) were performed for each decedent during surgery. In 14 (58%) cases, death occurred within 24 hours of surgery; the mean interval from procedure to death was 2.8 days (range = 0–18 days). Nine surgical clinics were linked to the deaths; two clinics were linked to two or more deaths.

### Cause of Death

Autopsy reports were available for 20 (83%) cases with medical records; all autopsy-confirmed deaths were attributed to intraoperative and postoperative complications. Fat embolism was the cause of death in 11 (55%) of 20 cases and pulmonary venous thromboembolism in seven (35%). 

## Discussion

A total of 93 U.S. citizens were reported to have died in the Dominican Republic during 2009–2022 soon after receiving cosmetic surgery. Medical records were available for 24 of 29 decedents during 2019–2020, most of whom were young to middle-aged women. Among the 20 fatalities during 2019–2020 with an autopsy report available for review, 18 (90%) deaths were attributed to embolic phenomena. A large proportion of decedents had personal (92%) or procedural (100%) risk factors for perioperative embolism. Among deaths due to fat emboli, all patients had undergone liposuction and gluteal fat transfer. Fat embolism is a recognized risk associated with fat injections, particularly as used in gluteal augmentation, a procedure in which fat is harvested from the patient and then injected into the buttocks to augment the body silhouette (*4*). Recommendations to avoid injecting the fat into the deep muscular layers of the buttocks to reduce the possibility of fat embolism have been previously published (*4*,*5*). Risk factors for venous thromboembolism in this report included BMI ≥25 kg/m^2^ (96% of patients), aged ≥40 years (50%), having undergone procedures on the trunk of the body (100%), or having undergone two or more procedures during the same operation (100%) (*6*–*8*). Preoperative ascertainment of patient risk for venous thromboembolism should be considered an expected standard of care during the preoperative evaluation and can be accomplished using validated risk-assessment models (e.g., Caprini score)** to help guide and incorporate the appropriate use of mechanical methods (e.g., early ambulation or compression devices) and chemoprophylaxis (e.g., anticoagulant and antithrombotic agents) to protect against periprocedural venous thromboembolism^††^ (*9*). The findings in this report highlight the importance of considering patient and operative risk factors when determining whether to proceed with elective cosmetic surgery.

### Limitations

The findings in this report are subject to at least three limitations. First, no reliable statistics on the number of U.S. citizens who receive cosmetic surgery in the Dominican Republic each year are available, precluding calculation of the risk for perioperative death. Second, this report might be an underestimate of the number of deaths among U.S. citizens receiving cosmetic surgery in the Dominican Republic because it only included deaths that were reported to the U.S. Embassy. Other investigators have documented adverse outcomes from cosmetic surgeries performed in the Dominican Republic that were only recognized after the patient had returned to the United States (*1*,*2*). Finally, perioperative deaths are rare complications of cosmetic surgery, and this report does not address other well-documented adverse events such as postsurgical infections that can result in substantial morbidity (*1*,*2*).

### Implications for Public Health Practice

Public health departments can make recommendations to improve medical care within their jurisdictions, and surveillance can identify new or ongoing health concerns. In 2019, the Dominican Republic MOH issued safety and quality recommendations to cosmetic surgeons, including training and licensure requirements, specific recommendations for patients to have cardiac and pulmonary evaluations before surgery, and that no more than two major procedures should be scheduled during one operation (*5*). After CDC shared preliminary results of the investigation of cosmetic surgery–associated deaths, the MOH distributed the safety and quality guidelines (*5*) to cosmetic surgeons in the country, made monitoring visits to 77 facilities that offered cosmetic surgery, and certified infection control committees for high volume cosmetic surgery centers. The MOH created a multilateral commission, including representatives from the Dominican Society of Plastic Surgery, to review perioperative adverse events, including deaths, and recommend disciplinary or administrative sanctions when warranted. Results of ongoing passive surveillance by the Dominican Republic government are provided to the U.S. Embassy regarding deaths of U.S. citizens after medical care.

As a result of this investigation, the U.S. State Department updated the Medical Tourism and Elective Surgery advisory on the website of the U.S. Embassy in the Dominican Republic^§§^ to provide a list of steps to take to reduce the risk for adverse outcomes, including a recommendation to obtain international travel insurance to cover medical evacuation back to the United States.

U.S. citizens considering cosmetic surgery abroad should consult with their primary health professionals about their inherent risk for adverse events after surgery and preventive measures they can take to reduce the risk. They should consult with a travel medicine specialist ≥1 month before travel, and, as air travel and surgery independently increase the risk for blood clots, patients should allow adequate time between flying to and from a destination for surgery to reduce the risk for complications (*10*).

## Footnotes

* These senior authors contributed equally to this report.

† The Dominican Republic MOH is aware of 70–80 clinics that provide plastic surgery; however, how many of these clinics provide plastic surgery to medical tourists, including to those who are U.S. citizens, is unknown. In addition, the number of private cosmetic surgeons who might operate outside of a clinic setting is unknown.

§ 45 C.F.R. part 46, 21 C.F.R. part 56; 42 U.S.C. Sect. 241(d); 5 U.S.C. Sect. 552a; 44 U.S.C. Sect. 3501 et seq.

¶ Overweight is defined as a BMI = 25.0–29.9 kg/m^2^; obesity is defined as a BMI ≥30 kg/m^2^. https://www.cdc.gov/obesity/basics/adult-defining.html

**
https://capriniriskscore.org/

†† https://pubmed.ncbi.nlm.nih.gov/37882602/; https://doi.org/10.1002/aorn.14019

§§
https://do.usembassy.gov/services/medical-assistance  **TABLE. Characteristics of perioperative deaths associated with cosmetic surgery procedures performed among U.S. citizens** ***** **(N = 24) — Dominican Republic, 2019–2020**
**Table:**

Characteristic	No. (%)		
	Total	2019	2020
**Total no. of decedents**	**24 (100)**	**10 (40)**	**14 (60)**
**Female sex**	**24 (100)**	10 (100)	14 (100)
**Mean BMI^†^ (range)**	**32 (24–44)**	31 (25–38)	32 (24–44)
**Mean age (range)**	**41 (26–61)**	40 (26–61)	41 (29–55)
**Risk factor for venous thromboembolism **			
BMI ≥25 kg/m^2^,^†^ no./No.	**22/23 (96)**	10/10 (100)	12/13 (92)
Aged ≥40 years	**12 (50)**	4 (40)	8 (57)
Diabetes mellitus	**2 (8)**	1 (10)	1 (7)
Current tobacco use	**3 (13)**	1 (10)	2 (14)
Current oral contraceptive use	**2 (8)**	0 (—)	2 (14)
Body (trunk) procedure^§^	**24 (100)**	10 (100)	14 (100)
≥2 procedures	**24 (100)**	10 (100)	14 (100)
≥3 procedures	**18 (75)**	5 (50)	13 (93)
**Preoperative evaluation documented**			
Cardiologist	**18 (75)**	8 (80)	10 (71)
Pulmonologist	**11 (46)**	4 (40)	7 (50)
**Procedure**			
Liposuction	**24 (100)**	10 (100)	14 (100)
Gluteal fat transfer	**22 (92)**	9 (90)	13 (93)
Abdominoplasty	**14 (58)**	4 (40)	10 (71)
Breast augmentation or reduction	**11 (46)**	3 (30)	8 (57)
No. of procedures performed, mean (range) per decedent	**3.0 (2–4)**	2.6 (2–4)	3.2 (2–4)
**Timing of death**			
Death within 24 hours	**14 (58)**	4 (40)	10 (71)
Interval from procedure to death, days, mean (range)	**2.8 (0–18)**	3.3 (0–12)	2.4 (0–18)
**Cause of death available from autopsy report**	**20 (83)**	8 (80)	12 (86)
Fat embolism	**11 (55)**	5 (63)	6 (50)
Pulmonary venous thromboembolism	**7 (35)**	2 (25)	5 (42)
Hemorrhagic shock	**1 (5)**	0 (—)	1 (8)
Sepsis^¶^	**1 (5)**	1 (13)	0 (—)

**Abbreviation:** BMI = body mass index.

* Information was available for 24 of 29 deaths that occurred in 2019 and 2020.

^†^ BMI missing for one record from 2020.

^§^ Body (trunk) procedures included abdominoplasty, liposuction, gluteal fat transfer, and breast augmentation or reduction.

^¶^ Due to intestinal perforation.
